# Thromboinflammation in coronavirus disease 2019: The clot thickens

**DOI:** 10.1111/bph.15594

**Published:** 2021-07-19

**Authors:** Raayma Iffah, Felicity N. E. Gavins

**Affiliations:** ^1^ Department of Life Sciences, Centre for Inflammation Research and Translational Medicine (CIRTM) Brunel University London London UK

**Keywords:** inflammation, neutrophils, platelets, resolution pharmacology, thromboinflammation, thrombosis

## Abstract

Since the start of the severe acute respiratory syndrome coronavirus 2 (SARS‐CoV‐2) pandemic, a disease that has become one of the world's greatest global health challenges, the role of the immune system has been at the forefront of scientific studies. The pathophysiology of coronavirus disease 2019 (COVID‐19) is complex, which is evident in those at higher risk for poor outcome. Multiple systems contribute to thrombosis and inflammation seen in COVID‐19 patients, including neutrophil and platelet activation, and endothelial dysfunction. Understanding how the immune system functions in different patient cohorts (particularly given recent emerging events with the Oxford/AstraZeneca vaccine) is vital to understanding the pathophysiology of this devastating disease and for the subsequent development of novel therapeutic targets and to facilitate possible drug repurposing strategies that could benefit society on a global scale.

**LINKED ARTICLES:**

This article is part of a themed issue on The second wave: are we any closer to efficacious pharmacotherapy for COVID 19? (BJP 75th Anniversary). To view the other articles in this section visit http://onlinelibrary.wiley.com/doi/10.1111/bph.v179.10/issuetoc

**Abbreviations**
AnxA1: annexin A1
ARDS: acute respiratory distress syndrome
C3: complement component 3
C4d: complement component C4d
C5b‐9: complement component C5b
CD8^+^ T cell: cytotoxic T cell
CoV: coronavirus
COVID‐19: coronavirus disease 2019
DAMPs: danger‐associated molecular patterns
factor IIa: blood coagulation factor IIa
GP: glycoprotein
GPIb: CD42, glycoprotein Ib
LMWH: low‐molecular‐weight heparin
Mac‐1: CD11b/CD18, macrophage 1
MASP‐2: mannose‐binding lectin‐associated serine protease‐2
MBL: mannose‐binding lectin
MERS‐CoV: Middle East respiratory syndrome coronavirus
MHRA: Medicines and Healthcare products Regulatory Agency
MPO‐DNA: myeloperoxidase*‐*DNA
NETs: neutrophil extracellular traps
PNAs: platelet–neutrophil aggregates
PSGL‐1: P‐selectin glycoprotein ligand‐1
RBD: receptor‐binding domain
RCTs: randomised controlled trials
S: transmembrane spike
SARS‐CoV: severe acute respiratory syndrome coronavirus
SARS‐CoV‐2: severe acute respiratory syndrome coronavirus 2
TF: tissue factor
TMPRSS‐2: transmembrane protease serine 2
UFH: unfractionated heparin
WHO: World Health Organisation

## 1 INTRODUCTION

Severe acute respiratory syndrome coronavirus 2 (SARS‐CoV‐2) is the aetiological agent of coronavirus disease 2019 (COVID‐19), a disease that has had global impact, with case rates surging to over 3000 deaths a day across Europe alone. Moreover, since the initial reported emergence in December 2019 in the Hubei Province of Wuhan, China, and the World Health Organisation (WHO) declaring the COVID‐19 outbreak a pandemic (11 March 2020), 128 million confirmed cases of COVID‐19 have been reported globally, including 2.8 million deaths. As of this year (2021), there have been profound breakthroughs such as the approval and administration of vaccines from Pfizer‐BioNTech, Moderna and Oxford/AstraZeneca, with additional vaccines from Valneva, CureVac Novavax and Johnson & Johnson currently under assessment by the Medicines and Healthcare products Regulatory Agency (MHRA), with others in the pipeline (e.g., GlaxoSmithKline/Sanofi Pasteur's vaccine, which may be available by the final quarter of 2021).

### 1.1 Coronaviruses

Coronaviruses (CoVs), of which there are four genera (alpha, beta, gamma and delta), are a family of enveloped, positive‐sense, single‐stranded and highly diverse RNA viruses (Zhu et al., 2020). Three betacoronaviruses have been identified to cause severe respiratory illness in humans including severe acute respiratory syndrome CoV (SARS‐CoV), Middle East respiratory syndrome CoV (MERS‐CoV) and SARS‐CoV‐2 (McFadyen et al., 2020). Although bats have been suggested to be the primary origin of SARS‐CoV and SARS‐CoV‐2, the precise origin and mechanism by how SARS‐CoV‐2 manifested in humans is still unclear, with other possible hosts (such as the Malayan pangolins [*Manis javanica*]), under investigation (Lam et al., 2020).

SARS‐CoV‐2 is a single‐stranded RNA virus, and entry into host cells initiates with their transmembrane spike (S) glycoproteins (GPs) encompassing S1 and S2 subunits. The S1 unit binds to host cell receptors, whereas the S2 subunit aids in viral and host cell fusion (Walls et al., 2020). Like SARS‐CoV, SARS‐CoV‐2 binds via the human ACE2, a cell surface protein with peptidase activity, expressed on the endothelial surface (Davidson et al., 2020; Hoffmann et al., 2020). In comparison with SARS‐CoV, SARS‐CoV‐2 has different amino acid residues within the receptor‐binding domain (RBD) of the S protein and a polybasic furin cleavage site at the junction of S1 and S2. In addition, transmissibility and pathogenicity of SARS‐CoV‐2 is linked to cellular proteases such as furin and the transmembrane protease serine 2 (TMPRSS‐2) (McFadyen et al., 2020). Although the functionality of these specific features of SARS‐CoV‐2 has yet to be fully elucidated, they could provide key information to the prothrombotic phenotype associated with SARS‐CoV‐2 and why patients with this specific CoV have higher thrombosis rates than SARS‐CoV and MERS‐CoV infections.

### 1.2 Clinical manifestations and thromboinflammation

A number of clinical manifestations such as fever, dry cough, malaise, sore throat, chest pain, dyspnoea, nausea, diarrhoea and vomiting have been associated with infection with SARS‐Cov‐2 (Tian et al., 2020). The spectrum of symptomatic infection ranges from mild to critical, although there remains uncertainty around the proportion of asymptomatic infections. Among hospitalised patients, the proportion of critical or fatal disease is higher, with many progressing to acute respiratory distress syndrome (ARDS), respiratory failure and eventually death (Bost et al., 2021). Interestingly, the ARDS associated with COVID‐19 patients differs from that caused by other infective or traumatic insults, with the ‘cytokine storm’ (i.e., increased cytokines released from the blood, which is associated with disease severity) being only partly involved in COVID‐19 patients (Maier et al., 2020). Furthermore, IL‐6 levels are 60‐fold to 90‐fold higher in ARDS patients compared with COVID‐19 patients, although the reasons for these differences are unknown. It is conceivable that SARS‐CoV‐2 takes over the host immune system, impairing antiviral immunity and triggering chronic inflammation by involving inflammatory cytokines (Bost et al., 2021; Sinha et al., 2020). Certainly, a hallmark of SARS‐CoV‐2 is the release of not only IL‐6 but also other cytokines and chemokines including IL‐1β, IL‐2, IL‐6, IL‐7, IL‐8, IL‐10, IL‐17, TNF‐α, CCL2, CCL3 and CXCL10, all of which correlate with adverse clinical outcomes. The inflammatory effects of cytokines also activate vascular endothelial cells, disrupting endothelial function and integrity, which leads to increased platelet and leukocyte recruitment, resulting in a pro‐inflammatory and prothrombotic state (Connors & Levy, 2020).

Autopsy reports from SARS‐CoV‐2 patients show multi‐organ dysfunction, with highest viral titres in the lungs and immune cells, and the presence of endothelial inflammation and cell death (Gu et al., 2005; Mazzulli et al., 2004). It is likely that hypoxia also promotes a prothrombotic endothelial phenotype in SARS‐CoV‐2 via hypoxia‐inducible transcription factor activation and endothelial tissue factor (TF) up‐regulation.

The scale of COVID‐19 severity also increases with co‐morbidities such as hypertension, chronic kidney disease, obstructive sleep apnoea, asthma diabetes and obesity (Tian et al., 2020). Patients with underlying cardiovascular disease are prone to increased severity of COVID‐19 and a fivefold increase in mortality (Yang et al., 2020). Therefore, managing and controlling cardiovascular risk factors is a high priority.

COVID‐19 is associated with a prothrombotic state, which can manifest as microvascular thrombosis, venous thromboembolism or arterial thrombosis (McFadyen et al., 2020). The cause of this prothrombotic state may relate to the appreciated link between thrombosis and inflammation (termed ‘thromboinflammation’) in which thrombosis can amplify inflammation and systemic inflammation can beget thrombosis. Although efforts to understand the complex immunological landscape in COVID‐19 are evolving, both platelet activation and platelet–neutrophil interactions play a crucial role in thromboinflammation.

## 2 ROLE OF NEUTROPHILS IN COVID‐19

Neutrophils are the immune system's first responders having crucial functions in immunity and repair. Upon activation, they produce pro‐inflammatory cytokines (including IL‐6, TNF‐α and IL‐1β), generate ROS and release haematopoietic serine proteases (neutrophil elastase, proteinase 3 and cathepsin G), microparticles and neutrophil extracellular traps (NETs). Neutrophils possess antimicrobial properties capable of not only killing both Gram‐positive and Gram‐negative bacteria, but they can also act as a double‐edged sword mediating tissue injury and perpetuating the inflammatory response (Kolaczkowska & Kubes, 2013). NETs, ROS and serine proteases can all independently, or collectively, up‐regulate thromboinflammatory processes.

**FIGURE 1:**
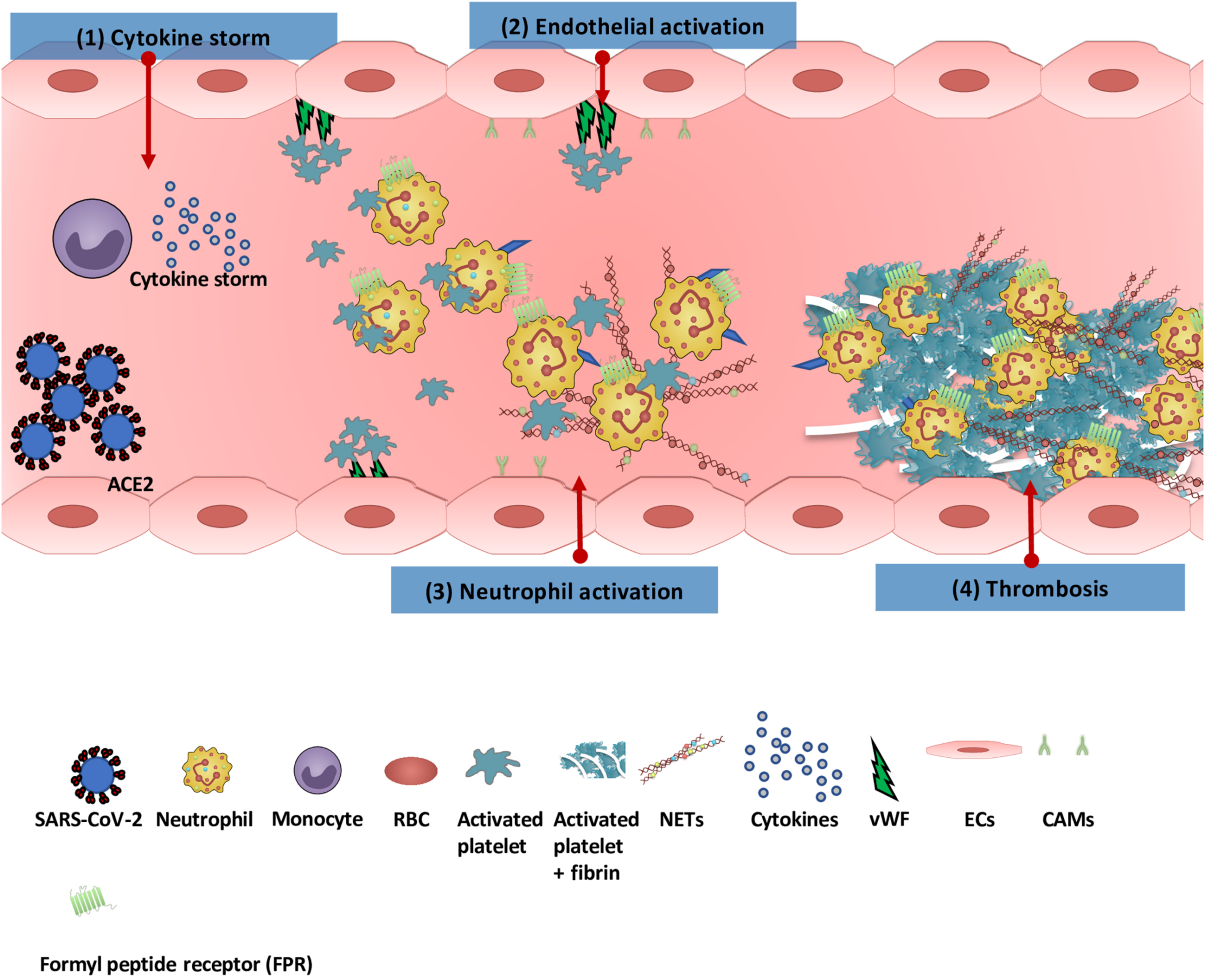
Thromboinflammation in coronavirus disease 2019 (COVID‐19). (1) Severe acute respiratory syndrome coronavirus 2 (SARS‐CoV‐2) binds via the ACE2 expressed on the endothelial surface. The virus activates monocytes releasing a cytokine storm (including IL‐6, IL‐1β, IL‐2, IL‐6, IL‐7, IL‐8, IL‐10, IL‐17, TNF‐α, CCL2, CCL3, and CXCL10 , all of which correlate with adverse clinical outcomes). (2) The inflammatory effects of cytokines also activate vascular endothelial cells (ECs), disrupting endothelial function and integrity, which leads to increased platelet and neutrophil recruitment. (3) Increased neutrophil at inflammatory site results in increased expression of adhesion molecules such as L‐selectin, P‐selectin and intercellular adhesion molecule‐1 on ECs. Activated neutrophils release their neutrophil extracellular traps (NETs, which contain a backbone of DNA and citrullinated histone [H3Cit], cathepsin G and neutrophil elastase [NE]), which trap platelets, increasing their activation and aggregation; this in turn further activates neutrophils to produce more NETs. (4) This cycle of events triggers coagulation cascade activation, which in turn increases fibrin formation and thrombosis. CAMs, cellular adhesion molecules; RBC, red blood cell; vWF, von Willebrand factorIFFAHANDGAVINS3

In the context of COVID‐19, neutrophilia signifies worse outcomes, with autopsy studies showing neutrophil infiltration in pulmonary capillaries, acute capillaries with fibrin deposition, extravasation of neutrophils in the alveolar space and neutrophil mucositis of the trachea (Gu et al., 2005; Yao et al., 2020). Bost et al. (2021) revealed at least 10 different neutrophil states present in blood and bronchoalveolar lavage of COVID‐19 patients, with resting state phenotype mainly associated with patients with mild disease, and activated or immature phenotype associated with patients with severe disease. These results suggest that COVID‐19 is associated with a state of ‘immune silence’ (demonstrated by loss of neutrophil and monocyte immunosuppression and the replacement of lung memory CD8^+^ T cells by naïve T cells), correlating with severe clinical manifestation and outcome (Bost et al., 2021). Diao et al. (2020) showed that T cells are dysfunctional with increased expression of exhaustion molecules related to heightened systemic inflammation, including IL‐6 levels. We have previously shown that IL‐6 plays a major role in T cell‐dependent thromboinflammatory responses to angiotensin II and in the activation and aggregation of platelets (Senchenkova, Russell, et al., 2019). These findings support the hypothesis that drug discovery programmes based on T cell‐dependent, IL‐6 signalling pathways may lead to protection against thromboinflammation in COVID‐19 (Figure 1).

As observed with other pathological conditions (e.g., sickle cell disease) (Ansari et al., 2021), NET levels are increased in COVID‐19 patients, with sera from COVID‐19 patients being shown to trigger NET release from control neutrophils and containing increased myeloperoxidase*‐*DNA (MPO‐DNA) complexes and citrullinated histone H3 levels, which correlated with disease severity (Veras et al., 2020; Zuo et al., 2020). These results suggest that NETs may contribute to COVID‐19 pathology and NET biomarkers may help to predict clinical worsening and venous thromboembolism.

Neutrophil degranulation and NET formation lead to various intracellular danger‐associated molecular patterns (DAMPs) activating pattern recognition receptors on nearby immune and non‐immune cells releasing pro‐inflammatory mediators (Tomar et al., 2020). DAMPs activate properdin, factor B and C3, all components of the alternative pathway necessary to induce the complement cascade. Reports have shown increased activation of the complement system in severe COVID‐19 patients (Holter et al., 2020). Lung biopsies from COVID‐19 patients have shown deposits of terminal complement components C5b‐9, C4d and mannose‐binding lectin (MBL)‐associated serine protease‐2 (MASP‐2), consistent with sustained, systemic activation of the complement pathways (Magro et al., 2020). These findings highlight the therapeutic strategy of complement targeted therapies for COVID‐19‐mediated thrombosis.

The initial neutrophil response also leads to interactions with platelets via a variety of different mechanisms including Mac‐1 (CD11b/CD18)/glycoprotein Ib (CD42) and P‐selectin/P‐selectin glycoprotein ligand‐1 (PSGL‐1), formation of fibrin cross‐links (via Mac‐1/fibrin interaction) and induction of extrinsic TF/factor IIa pathway, generating thrombin (De Meyer et al., 2016). TF‐enriched NETs and a high neutrophil count are associated with increased disease severity and poor prognosis in COVID‐19 (Skendros et al., 2020), amplifying the need for increased research regarding platelet–neutrophil interactions in thrombogenesis.

## 3 ROLE OF PLATELETS AND IN COVID‐19

Platelets are traditionally known for their role in haemostasis and formation of thrombi. However, they are increasingly being recognised as key effector cells in inflammation, influencing innate and adaptive immune responses (Senchenkova, Ansari, et al., 2019). Migration of single platelets acts as mechanosensors to collect and bundle bacteria for neutrophil phagocytosis (Gaertner et al., 2017), although their uncontrolled activation can result in pathogenic thrombosis. Platelets from COVID‐19 patients show increased aggregation, adhesion and spreading on fibrinogen and collagen (via up‐regulation of the MAPK pathway) and increased activity of thromboxane A_2_ (marker of platelet activation) (Manne et al., 2020).

Thrombocytopenia in COVID‐19 is common, with meta‐analysis demonstrating a link with a fivefold increased risk of severe disease (Lippi et al., 2020). Thrombocytopenia has also been linked with issues of rare blood clotting events, which are now associated with the Oxford/AstraZeneca vaccine (ongoing pharmacovigilance will determine outcome), with seven deaths being reported in the 30 cases reported in the United Kingdom alone (at the time of writing this review, evidence is shifting towards a causal link between the vaccine and rare blood clots).

Elevated d‐dimers, fibrinogen and von Willebrand factor levels are also associated with a higher mortality rate. Autopsies of cardiac and pulmonary tissue from COVID‐19 patients have shown the presence of megakaryocytes (Rapkiewicz et al., 2020). A recent study by Manne et al., using platelet RNA sequencing, demonstrated COVID‐19‐induced significant changes in platelet transcriptome and proteome, and platelet hyperreactivity (Manne et al., 2020), which may contribute to COVID‐19 pathophysiology by increasing platelet homotypic and heterotypic interactions. During infection, platelets became hyperactive as shown by the increased surface P‐selectin expression and increased formation of circulating platelet–neutrophil aggregates (PNAs) via binding with PSGL‐1 (Manne et al., 2020). The generation of PNAs recruit neutrophils to damaged lung capillaries. Thrombotic events in COVID‐19 may be attributed to platelets augmenting inflammation through the generation of NETs and pro‐coagulant platelets, increased aggregates (e.g., PNAs) and the release of bioactive substances. A greater understanding of the role that neutrophils and platelets play in thromboinflammation and their involvement in the pathophysiology of COVID‐19 is needed for drug discovery focusing on dampening thromboinflammatory processes.

## 4 THERAPEUTIC INTERVENTION THROMBOINFLAMMATION RESOLUTION

Understanding neutrophil and platelet responses in the context of thromboinflammation in COVID‐19 has resulted in promising preclinical studies demonstrating that, for example, immune regulatory properties are being lost in patients with COVID‐19, with the virus seemingly to ‘suppress’ or ‘silence’ the immune system (Bost et al., 2021). Drugs that can ‘stimulate’ or ‘reawaken’ the immune system may be the way forward, as seen with dexamethasone (Bost et al., 2021; The RECOVERY Collaborative Group et al., 2021). Other therapies may lie in targeting the cytokine storm with anti‐inflammatory agents such as IL‐6 and IL‐1β antagonists.

Accumulating data linking inflammation and thrombosis supports the hypothesis that anti‐inflammatory therapies may limit thrombosis and that antithrombotic therapies may reduce vascular inflammation (Vital et al., 2016). Due to the high incidence of thrombotic events seen in severe stages of COVID‐19, most clinical guidelines agree to the administration of low‐molecular‐weight heparin (LMWH), as thrombo‐prophylaxis, to hospitalised COVID‐19 patients. However, the correct dose for hospitalised patients and thromboprophylaxis approach to ambulant patients remains controversial. A wide range of antithrombotic therapies are currently being assessed in ongoing and recent randomised controlled trials (RCTs) directed at outpatients, hospitalised patients and critically ill patients with COVID‐19. These trials involve the use of antiplatelet agents, anticoagulants and fibrinolytic agents or a combination (Table 1). Current antithrombotic therapies consist of various interventions including thromboprophylaxis with unfractionated heparin (UFH), or LMWH; an intensive thromboprophylaxis protocol with LMWH, antithrombin, clopidogrel and salvage therapy with tissue plasminogen activator and heparin (Maldonado et al., 2020).

**TABLE 1:** Clinical trials examining the potential of antithrombotic therapies for the treatment of COVID‐19‐related side effects

Trial name	Agent	ClinicalTrials.gov identifier	Summary
Antiplatelet agents			
REMAP‐CAP	Aspirin or P2Y_12_ receptor antagonists	NCT02735707	7100 patients to receive multiple therapeutic interventions; anticoagulant arm and antiplatelet agent arm evaluating aspirin and P2Y_12_ receptor antagonists clopidogrel, ticagrelor or prasugrel.
PEAC	Aspirin	NCT04365309	Aims to test efficacy of aspirin in shortening clinical recovery time.
RECOVERY	Aspirin	—	Investigation of the effects of aspirin on all‐cause mortality among hospitalised patients with 20,000 participants.
RESIST	Aspirin and atorvastatin	CTRI/2020/07/026791	Evaluation of the role of aspirin and atorvastatin in clinical deterioration by progression in accordance with the WHO clinical improvement ordinal score in 800 hospitalised patients.
CAM‐COVID‐19	Aspirin	—	Evaluating the impact of a higher dose of aspirin in 34 patients (325 mg four times a day) in addition to colchicine and montelukast on inflammatory markers such as high‐sensitivity C‐reactive protein.
PARTISAN	Prasugrel	NCT04445623	Comparing the effect of prasugrel with placebo among 128 patients on the primary outcome of improved oxygen expressed as the PaO_2_/FiO_2_ ratio at 7‐day follow‐up.
Antithrombotic drugs			
DAWn‐Antico	LMWH or UFH	—	A randomised, open‐label, adaptive, proof‐of‐concept clinical trial of modulation of host thromboinflammatory response in patients with COVID‐19. Comparison of low MW heparins at 50‐IU anti‐Xa per kg twice daily—or 75‐IU anti‐Xa twice daily for ICU patients—in combination with aprotinin to standard thromboprophylaxis in hospitalised COVID‐19 patients.
X‐COVID‐19	LMWH or UFH	—	—
COVID‐19 HD	LMWH or UFH	—	RCT comparing efficacy and safety of high vs. low LMWH dosages in hospitalised patients with severe COVID‐19 pneumonia and coagulopathy not requiring invasive mechanical ventilation.
COVI‐DOSE	LMWH or UFH	—	Weight‐adjusted vs. fixed low doses of LMWH for venous thromboembolism prevention in COVID‐19.
EMOS‐COVID	LMWH or UFH	NCT04360824	Testing efficacy and safety of prophylactic anticoagulation therapy in hospitalised patients.
Direct oral anticoagulants (DOACs)			
ACTION	Rivaroxaban	NCT04394377	RCT to evaluate a routine full anticoagulation strategy in patients with coronavirus compared with usual SOC with prophylactic anticoagulation involving 20‐mg·day^−1^ rivaroxaban followed by enoxaparin/UFH vs. control group with 40‐mg·day^−1^ enoxaparin.
COVID‐PREVENT	Rivaroxaban	—	A study investigating the effect of anticoagulation therapy on clinical outcomes in COVID‐19.
FREEDOM	Edoxaban	NCT04512079	A multicentre, open‐label RCT determining effectiveness and safety of enoxaparin and apixaban in patients hospitalised (but not yet intubated) with COVID‐19.
XACT	Rivaroxaban	NCT04640181	A multicentre RCT studying the potential benefit of treatments with a direct Factor Xa inhibitor (rivaroxaban) compared with SOC dose subcutaneous LMWH (Lovenox) in hospitalised subjects with COVID‐19.
Tissue plasminogen activator (tPA)			
AtTAC	Alteplase	NCT04453371	tPA treatment for an atypical ARDS: Microvascular COVID‐19 Lung Vessels Obstructive Thromboinflammatory Syndrome (MicroCLOTS).
STARS	Alteplase	NCT04357730	Fibrinolytic therapy to treat ARDS in the setting of COVID‐19 infection.
TRISTRADS	Alteplase	NCT04640194	ThRombolysIS therapy for ARDS. A Phase IIb/III operationally seamless, open‐label, randomised, sequential, parallel‐group adaptive study to evaluate the efficacy and safety of daily intravenous alteplase treatment given up to 5 days on top of SOC compared with SOC alone, in patients with ARDS triggered by COVID‐19.
TACOVID	Tenecteplase	NCT04505592	A placebo‐controlled, double‐blind, randomised Phase II dose‐escalation study intending to study the safety and efficacy of tenecteplase for the treatment of COVID‐19‐associated respiratory failure. Hypothesis is that the administration of drug with heparin anticoagulation will improve patients' clinical outcome.

Abbreviations: ARDS, acute respiratory distress syndrome; COVID‐19, coronavirus disease 2019; ICU, intensive care unit; LMWH, low‐molecular‐weight heparin; RCT, randomised controlled trial; SOC, standard of care; UFH, unfractionated heparin; WHO, World Health Organisation.

There is a growing evidence of the importance of resolution biology in vascular inflammation to develop innovative approaches for the treatment of diseases, which may include COVID‐19. Inflammation resolution is an active process involving specific endogenous mediators (e.g., annexin A1 [AnxA1] and aspirin‐triggered lipoxin A_4_ ) and pathways (e.g., formyl peptide receptor 2 [FPR2/ALX] pathway) (Senchenkova, Ansari, et al., 2019). FPR2 agonists are being developed and currently tested in man. In the context of thromobinflammation and the possible insights for therapeutic strategies for COVID‐19 treatments, we recently discovered that targeting the AnxA1/Fpr2/ALX pathway promotes thromboinflammation resolution by altering both the platelet phenotype (from pro‐pathogenic to regulatory) (Senchenkova, Ansari, et al., 2019) and the pathological neutrophil phenotype (from a pro‐NETotic to pro‐apoptotic) (Ansari et al., 2021).

## 5 CONCLUSION

COVID‐19 is a devastating disease that has affected the United Kingdom and our global community in unprecedented ways. The pathophysiology is complex with many systems likely to contribute to the prothrombotic state including inflammation, platelet and neutrophil activation, endothelial cell dysfunction, NETs and complement factors. However, the mechanism that drives COVID‐19‐associated thromboinflammation has not yet been fully elucidated and there is an urgent unmet clinical need to fully understand and characterise this disease. These discoveries will unearth ways to develop pharmacological strategies, which may also focus on resolution biology.

### 5.1 Nomenclature of targets and ligands

Key protein targets and ligands in this article are hyperlinked to corresponding entries in the IUPHAR/BPS Guide to PHARMACOLOGY (http://www.guidetopharmacology.org) and are permanently archived in the Concise Guide to PHARMACOLOGY 2019/20 (Alexander, Christopoulos et al., 2019; Alexander, Fabbro, et al., 2019a, b).

## CONFLICT OF INTEREST

The authors declare no conflicts of interest.

## Data Availability

No data have been shared.
